# *Not Just a Pot*: Visual Episodic Memory in Cannabis Users and Polydrug Cannabis Users: ROC and ERP Preliminary Investigation

**DOI:** 10.3389/fnhum.2021.677793

**Published:** 2021-06-11

**Authors:** Alicja Anna Binkowska, Natalia Jakubowska, Maciej Gaca, Natalia Galant, Agnieszka Piotrowska-Cyplik, Aneta Brzezicka

**Affiliations:** ^1^SWPS University of Social Sciences and Humanities, Warsaw, Poland; ^2^Polish-Japanese Academy of Information Technology, Warsaw, Poland; ^3^Nencki Institute of Experimental Biology PAS, Warsaw, Poland; ^4^Institute of Forensic Genetics, Bydgoszcz, Poland; ^5^Institute of Food Technology of Plant Origin, Poznań University of Life Sciences, Poznań, Poland; ^6^Department of Neurosurgery, Cedars-Sinai Medical Center, Los Angeles, CA, United States

**Keywords:** cannabis (marijuana), polydrug use, recognition memory, EEG, late parietal component, SDT, ROC

## Abstract

**Background** While research has consistently identified an association between long-term cannabis use and memory impairments, few studies have examined this relationship in a polydrug context (i.e., when combining cannabis with other substances).

**Aims:** In this preliminary study, we used event-related potentials to examine the recognition process in a visual episodic memory task in cannabis users (CU) and cannabis polydrug users (PU). We hypothesized that CU and PU will have both–behavioral and psychophysiological–indicators of memory processes affected, compared to matched non-using controls with the PU expressing more severe changes.

**Methods** 29 non-using controls (CG), 24 CU and 27 PU were enrolled into the study. All participants completed a visual learning recognition task while brain electrical activity was recorded. Event-related potentials were calculated for familiar (old) and new images from a signal recorded during a subsequent recognition test. We used receiver operating characteristic curves for behavioral data analysis.

**Results** The groups did not differ in memory performance based on receiver operating characteristic method in accuracy and discriminability indicators nor mean reaction times for old/new images. The frontal old/new effect expected from prior research was observed for all participants, while a parietal old/new effect was not observed. While, the significant differences in the late parietal component (LPC) amplitude was observed between CG and PU but not between CG and CU nor CU and PU. Linear regression analysis was used to examine the mean amplitude of the LPC component as a predictor of memory performance accuracy indicator. LPC amplitude predicts recognition accuracy only in the CG.

**Conclusion** The results showed alterations in recognition memory processing in CU and PU groups compared to CG, which were not manifested on the behavioral level, and were the most prominent in cannabis polydrug users. We interpret it as a manifestation of the cumulative effect of multiple drug usage in the PU group.

## Introduction

Cannabis is the most commonly used illicit substance in the world (United Nations Office on Drugs & Crime (UNODC), 2019) and approximately 26% of adult Europeans have a history of using cannabis (European Monitoring Centre for Drugs and Drug Addiction (EMCDDA), 2018). And it is not without influence on their cognitive functioning. The most consistent and prominent acute effects of cannabis are impairments in the verbal learning and memory and working memory as shown in recent meta-analysis, where reported effect sizes were medium, between 0.5 and 0.7 (Zhornitsky et al., 2021). Moreover, impairments in various cognitive functions have been associated with the chronic cannabis use (lasting beyond intoxication phase), with the most consistent evidence in impairment of verbal episodic and working memory (Broyd et al., 2016; Curran et al., 2016; Figueiredo et al., 2020). Meta-analyses concentrated on the potential long-term effects of cannabis use on cognitive domains, has shown the significant deficits in executive functions, learning, working memory, attention, processing speed, and the estimated effect sizes were small (Grant et al., 2003; Schreiner and Dunn, 2012; Scott et al., 2018; Figueiredo et al., 2020; Kroon et al., 2020a).

Less is known about the long-term impact of cannabis on visual memory and learning. Studies suggest that THC (*tetrahydrocannabinol–*the main psychoactive compound of cannabis) may interfere with visual information processing, which can lead to impairment of the visual memory of objects and increase the chances of false recognition (Böcker et al., 2010; Winton-Brown et al., 2011). Recently conducted study has shown that people directly under the influence of cannabis exhibit impairment in visual episodic memory, including learning process (Doss et al., 2020). The question arises whether this effect persists in regular cannabis users when they are no longer directly intoxicated and if it is accompanied by altered brain function. The formation and retrieval in episodic memory involve mostly medial temporal lobe (MTL) structures such as the hippocampus and parahippocampal gyrus (Brewer et al., 1998; Desgranges et al., 1998). It is important to note that the highest density of cannabinoid receptors (CB1) is in the hippocampus and prefrontal cortex (Herkenham et al., 1990; Ameri, 1999).

There is strong evidence coming from studies on the relation between brain function and episodic memory, that event-related potential (ERP) components are helpful in understanding physiological correlates of episodic memory (Rugg and Curran, 2007; Hoppstädter et al., 2015). The majority of studies on learning and recognition tasks show that ERPs evoked by previously experienced stimuli (“old” or “familiar”) are more positive than ERPs evoked by new stimuli (“new”)–this effect is known in the literature as the so called “Old/New effect” (Rugg and Curran, 2007; Hoppstädter et al., 2015). There are two main components of the Old/New effect–the frontal negative (FN) deflection peaking between 300 and 500 ms, labeled the FN400, which is related to the familiarity process (Curran, 2000; Paller et al., 2007) and late positive component (LPC), which is a posterior positive deflection, peaking between 400 and 800 ms, related to the recollection process (Curran and Cleary, 2003; Rugg and Curran, 2007).

While there is still a discussion about the cognitive mechanisms involved in LPC generation, the research suggests that it is related to categorical response, decision accuracy, maintenance of visual working memory representations and memory match, despite recognition memory (Danker et al., 2008; Schendan and Maher, 2009; Gunseli et al., 2014). Study conducted by Finnigan et al. (2002) has shown that the LPC amplitude was significantly more positive for correct than incorrect recognition responses not only when old but also when new items were considered. Another research based on drift-diffusion modeling of behavioral data showed that the LPC amplitude predicts participants’ accuracy of recognition-memory decisions on a trial-by-trial basis (Ratcliff et al., 2016).

To the best of our knowledge, there is only one study investigating ERPs in non-intoxicated regular cannabis users and heavy alcohol drinkers, which used the verbal memory task (the Rey Auditory Verbal Learning Test, RAVLT) conducted by Smith et al. (2017). The results did not show any behavioral impairments, but indicated alterations in recognition memory processing manifested by a larger LPC in heavy drinkers compared to a control group. Cannabis-related differences were related to a smaller FN400 component and a lack of the Old/New effect, usually observed in a LPC response.

Detailed analysis of our collected data on self-reported substance use as well as the hair sample analysis on drug use (results were delivered after study accomplishment) revealed that the majority of recruited cannabis users actually use other illicit psychoactive substances as well.

Cannabis is the most commonly used drug within a polydrug context (Mitchell and Plunkett, 2000; Carlson et al., 2005; Lynskey et al., 2006; World Health Organisation, 2016). The majority of cannabis users do not restrict substance use to cannabis, but use other illicit or licit psychoactive substances (mainly alcohol). As far as we know, research including polydrug users (cannabis use only vs. combining cannabis with other illicit substances) is extremely rare. Such comparison offers a deeper insight into neurocognitive functioning of cannabis users and polydrug cannabis users. That is why we decided to include two cannabis users groups in our study–cannabis users and polydrug users (cannabis + 1 ≤ other illicit drugs). Alcohol and tobacco consumption level were controlled among groups as a potential confounding variable. As we were interested in the residual impact of cannabis, we included users who were not acutely intoxicated during the time of study and who have had at least 12 h abstinence from cannabis.

The objective of the current study was to investigate if visual episodic memory impairment lasts beyond the intoxication phase in regular cannabis users (residual effect) and in those with cannabis polydrug use, and if there are differences between users and the controls in electrical brain activity related to memory recognition. We decided to use sensitive analyses methods of memory performance based on signal detection theory (SDT) as they provide reliable information on memory qualities and are considered as an indicator of MTL-dependent declarative memories successful formation (Wais et al., 2006).

The primary aim of our study was to answer the following research questions: First, is performance on a visual episodic memory task affected in regular cannabis users compared to non-using controls? Second, are there differences in ERPs components related to recognition between these users and the controls? If this is the case, is there any relationship between electrophysiological and behavioral indicators of memory performance? Additionally, in line with previous research, we expected to observe the frontal (FN400) and parietal (LPC) Old/New effect. We also expected to find more positive LPC amplitude for correct than incorrect answers, which would predict memory accuracy.

## Materials and Methods

### Participants

Eighty-two participants provided informed consent to take part in this study, 79 were included in study (reasons for exclusion of three participants are described below), while 64 were included in EEG data analysis (15 participants were excluded because of bad EEG signal). The research protocol was approved by the SWPS University Research Ethics Committee. We included in study 29 non-using controls (CG) who used cannabis on fewer than two occasions a year, and had not used in the preceding 90 days and 50 cannabis users, that were further divided in two subgroups which consist of 24 cannabis users (CU) using cannabis at least once a month (regular use) for at least 2 years (long-term use), and 26 cannabis polydrug users (PU) defined as using cannabis (at least once a month for at least 2 years) and using at least one other illicit drug in the last 3 months. Inclusion criteria for all participants were as follows: 21–42 years of age; Polish as a first language; normal or corrected to normal vision. Participants were excluded if they self-reported a history of brain injury, diagnosis of neurological disease, psychotropic medications usage. Additional criteria for cannabis users included: using cannabis at least once a month (regular use) for at least 2 years (long-term use) and negative results in screening test for cannabis use disorder (measured as ≤12 points at The Cannabis Use Disorder Identification Test–Revised (CUDIT-R; Adamson et al., 2010) delivered in an online recruitment questionnaire before study. CUDIT-R is often used in academic settings and is consider as having good psychometric properties and time-efficient measurement (Adamson et al., 2010; Kroon et al., 2020b). It is important to note that we invited to our study participants who declared cannabis use only (and no other illicit drugs) while recruiting to study, however, analyses of collected data in lab settings and hair sample analyses revealed polydrug use patterns in half of cannabis users. That is why we decided to include them in a study as a separate group which constitute a representative sample of cannabis users (Mitchell and Plunkett, 2000; Carlson et al., 2005; Lynskey et al., 2006; World Health Organisation, 2016).

Participants were screened for diagnosed psychiatric disorders based on self-declaration of the presence of a diagnosis by a mental health specialist, eight participants reported depression or anxiety (4 CG, 1CU, and 3 PU), all other participants reported no psychiatric disorders. Table 1 shows demographic and substance use characteristics for 79 participants included in behavioral analyses, while Table 2 shows the same characteristics for 64 participants included in EEG data analyses.

**TABLE 1:** Demographic, neuropsychological and substance use characteristics for participants included in behavioral analyses.

**Group (*n* = 79)**	**Controls (*n* = 29)**	**Cannabis users (*n* = 24)**	**Polydrug users (*n* = 26)**	**Three group**	
				**comparisons**	
				F_2, 76_	*P*
**Male/female, %**	48.3/51.7	54.2/45.8	65.4/34.6		
**Age, years, mean (SD)**	28 (5.13)	29.29 (5.09)	27.67 (4.6)	0.611^a^	0.545
**Highest level of education (Years)**	16.8 (1.85)	17 (2.23)	16.3 (2.04)	0.737^a^	0.482
**IQ test scores**					
[1pt] WAIS scores Vocabulary	13.7 (2.06)	13.1 (1.72)	13.2 (1.7)	1.086^a^	0.343
WAIS scores Similarities	13 (1.92)	13.3 (2.22)	12.9 (1.81)	0.259^a^	0.773
WAIS scores Block design	12.9 (2.74)	13.4 (2.65)	13.6 (2.84)	0.441^a^	0.645
WAIS scores Matrix reasoning	12.9 (2.3)	13.2 (2.54)	13.2 (2.22)	0.196^a^	0.822
WAIS scores Digit span	12.1 (3.08)	13.1 (2.83)	13.2 (3.11)	1.086^a^	0.343
**Diagnosed psychiatric disorders (% in group)**	13.8	4.2	11.5		
**Alcohol, standard drinks per week, %**					
0	3.4	0	0		
<1	44.8	50	46.2		
1–3	37.9	45.8	38.5		
4–6	13.8	4.2	11.5		
7–14	0	0	3.9		
14≤	0	0	0		
**Tobacco, %**					
No	75.9	45.8	65.4		
Occasionally	24.1	45.8	26.9		
Regularly	0	8.3	7.7		
**Cannabis use pattern**					
**Onset age, years**	–	19.96 (5.7)	19.7 (3.43)		
**Duration, years**	–	9.04 (7.09)	8.61 (4.48)		
**Frequency of cannabis use (% of subjects) Lifetime**					
0	82.8	0	0		
Less than twice a year	17.2	0	0		
2–3 times per month	0	0	7.7		
1–3 times per week	0	33.3	23.1		
3–6 times per week	0	33.3	23.1		
Daily	0	33.3	38.7		
No answer	0	0	7.6		
**Frequency of cannabis use within past 30 days**					
0	100	4.2	7.7		
2–3 times per month	0	8.3	3.8		
1–3 times per week	0	33.3	26.9		
3–6 times per week	0	29.2	30.8		
Daily	0	25	30.8		
**Dose in grams per week (%)**				
Less than 1 g	0	33.3	30.8	
1–2 grams	0	41.7	23.1	
3–5 grams	0	25	30.8	
>5 grams	0	0	11.5	
No answer	0	0	3.8	
**Dose in puffs per one use**	–	7.5 (3)	7 (2.7)	
**Time since last cannabis use (%)**				
<12 h	0	8.3	7.7	
12–24 h	0	41.7	50	
1–3 days	0	16.7	26.9	
3–7 days	0	20.8	7.7	
7–14 days	0	4.2	0	
>14 days ago	0	8.3	3.8	
**Other illicit drug use in last 30 days (% of subjects)**				
0	100	100	61.5	
1 time per month	0	0	34.6	
2 < per month	0	0	3.8	
**Hair sample pos (number)**				
THC	–	12	19	
MDMA	–	–	17	
LSD	–	–	1	
Amphetamine	–	–	4	
Methcathinone	–	–	2	
Cocaine	–	–	8	
Cathine	–	–	1	

*Note: ^*a*^One-way ANOVAs; There were no significant differences between cannabis users (CU), polydrug users (PU), and control group (CG) in tobacco H(2) = 5.441, *p* = 0.066 and alcohol use patterns H(2) = 0.4, *p* = 0.819 as Kruskal–Wallis *H* tests revealed, gender X(2) = 1.659, *p* = 0.436, diagnosed psychiatric disorders X(2) = 3.256, *p* = 0.516 as χ^2^-tests shown. Comparison between CU and PU on cannabis use pattern shown no significant differences in: onset age *t*(44) = 0.19, *p* = 0.852, duration *t*(44) = 0.249, *p* = 0.805, dose in puffs per one use *t*(43) = 0.587, *p* = 0.561 (series of *t*-tests has shown), lifetime frequency of use *Z* = −0.326, *p* = 0.745, frequency of use in last 30 days *Z* = −0.403, *p* = 0.687, dose in grams per week *Z* = −1.050, *p* = 0.294 and time since last cannabis use *Z* = −1.044, *p* = 0.279 (series of Mann–Whitney *U* tests). It is important to note that the ordinal data on substance use are presented in table in percentages, however, during analyses (while performing series of Mann–Whitney *U* or Kruskal–Wallis *H* tests) they were coded numerically. Hair sample analyses time-window was 3 months preceding study participation.*

**TABLE 2:** Demographic, neuropsychological and substance use characteristics for participants included in event-related potential (ERPs) analyses.

**Group (*n* = 64)**	**Controls (*n* = 24)**	**Cannabis users (*n* = 19)**	**Polydrug users (*n* = 21)**	**Three group**	
				**comparisons**	
				F_2, 61_	*P*
**Male/female, %**	50/50	52.6/47.4	66.7/33.3		
**Age, years, mean (SD)**	28.3 (5.34)	28.9 (5)	27.9 (4.34)	0.246^a^	0.783
**Highest level of education (Years)**	16.8 (1.86)	16.8 (2.26)	16.3 (2.1)	0.42^a^	0.659
**IQ test scores**					
WAIS scores Vocabulary	13.8 (2.1)	13 (1.73)	13.4 (1.77)	0.933^a^	0.399
WAIS scores Similarities	13.1 (2.03)	13.2 (2.46)	13 (1.87)	0.032^a^	0.969
WAIS scores Block design	12.8 (2.95)	13.3 (2.68)	13.4 (2.71)	0.317^a^	0.73
WAIS scores Matrix reasoning	12.5 (2.28)	13.4 (2.36)	13 (1.94)	0.865^a^	0.426
WAIS scores Digit span	12.6 (2.64)	12.8 (3.08)	12.6 (3.12)	0.046^a^	0.955
**Diagnosed psychiatric disorders (% in group)**	16.7	5.7	4.8		
**Alcohol, standard drinks per week, %**					
0	4.2	0	0		
<1	41.7	47.4	47.6		
1–3	41.7	52.6	38.1		
4–6	12.5	0	14.3		
7–14	0	0	0		
14≤	0	0	0		
**Tobacco, %**					
No	75	47.4	61.9		
Occasionally	25	42.1	28.6		
Regularly	0	10.5	9.5		
**Cannabis use pattern**					
**Onset age, years**	–	19.47 (5.95)	20.06 (3.56)		
**Duration, years**	–	9.47 (7.73)	8.28 (3.97)		
**Frequency of cannabis use (% of subjects) Lifetime**					
0	87.5	0	0		
Less than twice a year	12.5	0	0		
2–3 times per month	0	0	9.5		
1–3 times per week	0	36.8	19		
3–6 times per week	0	26.3	28.6		
Daily	0	36.8	33.3		
No answer	0	0	9.6		
**Frequency of cannabis use within past 30 days**					
0	100	0	9.5		
2–3 times per month	0	10.5	4.8		
1–3 times per week	0	36.8	23.8		
3–6 times per week	0	26.3	38.1		
Daily	0	26.4	23.8		
**Dose in grams per week (%)**				
Less than 1 g	0	31.6	33.3	
1–2 grams	0	36.8	23.8	
3–5 grams	0	31.6	33.3	
>5 grams	0	0	4.8	
No answer	0	0	4.8	
**Dose in puffs per one use**	–	8.2 (2.6)	6.6 (2.7)	
**Time since last cannabis use (%)**				
<12 h	0	10.5	4.8	
12–24 h	0	42.1	52.4	
1–3 days	0	15.8	23.8	
3–7 days	0	21.1	10	
7–14 days	0	5.3	0	
>14 days ago	0	5.3	5	
**Other illicit drug use in last 30 days (% of subjects)**				
0	100	100	57.1	
1 time per month	0	0	42.9	
2 < per month	0	0	0	
**Hair sample pos (number)**				
THC	–	10	15	
MDMA	–	–	15	
LSD	–	–	1	
Amphetamine	–	–	4	
Methcathinone	–	–	1	
Cocaine	–	–	7	
Cathine	–	–	0	

*Note: ^*a*^One-way ANOVAs; There were no significant differences between CU, PU, and CG in tobacco H(2) = 3.459, *p* = 0.117 and alcohol use patterns H(2) = 0.837, *p* = 0.242 as Kruskal–Wallis *H* tests revealed, gender X(2) = 1.408, *p* = 0.495, diagnosed psychiatric disorders X(2) = 2.406, *p* = 0.3 as χ^2^-tests shown. Comparison between CU and PU on cannabis use pattern shown no significant differences in: onset age *t*(35) = −0.359, *p* = 0.722, duration *t*(35) = 0.587, *p* = 0.561, dose in puffs per one use *t*(34) = 1.832, *p* = 0.076 (series of *t*-tests has shown), lifetime frequency of use *Z* = −0.566, *p* = 0.571, frequency of use in last 30 days *Z* = −0.028, *p* = 0.978, dose in grams per week *Z* = −0.282, *p* = 0.788 and time since last cannabis use *Z* = −0.511, *p* = 0.609 (series of Mann–Whitney *U* tests). It is important to note that the ordinal data on substance use are presented in table in percentages, however, during analyses (while performing series of Mann–Whitney *U* or Kruskal–Wallis *H* tests) they were coded numerically. Hair sample analyses time-window was 3 months preceding study participation.*

Cannabis users were included to CU group if they reported in self-assessment regular and long-term cannabis use (described above) and hair sample analysis reflected no other drug metabolites detected in their hair [from analysis of hair samples reflecting past 3-months exposure: THC+ (*n* = 12); no cannabinoid metabolites detected (*n* = 12)]. Cannabis polydrug users were included in PU group, if they reported in self-assessment regular and long-term cannabis use and hair sample analysis reflected other drug metabolites [from analysis of hair samples reflecting past 3-months exposure: THC+ (*n* = 19); 1 ≤ other illicit drug metabolites detected (*n* = 26)]. The most popular drug used in PU was MDMA (*n* = 17). Non-drug using controls (CG) reported cannabis use on fewer than two occasions a year, no use in the preceding 90 days (and no other illicit drug use) in self-assessment and had no drug metabolites detected in hair samples. Hair samples were not collected from eight participants from the non-drug using control group.

As mentioned before three participants were excluded from all analyses (1 in CG and 1 in PU because of current use of psychotropic medication and another one in PU because of deviant results – performance at random level indicating no engagement in task, described in “Behavioral Performance” section and psychotropic medication use). 15 participants were excluded from EEG data analyses due to technical problems with recording. Four participant reported shorter that 12 h abstinence since last cannabis use (2 PU and 2 CU), while it was highly possible that they used cannabis at night preceding experimental sessions, we decided to include them in behavioral and EEG analyses (2 CU and 1 PU, one of them was excluded because of bad EEG signal), controlling whether the results would change in case of their absence.

Participants were recruited via advertisements and social media and received the description of their IQ test score and a sample of their brains’ electrical activity for their participation.

### Substance Use Assessment

Substance use was assessed by the self-reported drug history questionnaire, which included the age of when cannabis use first started, years of usage, days per month of usage, dose in grams per week and time since last use. Questions about other drug use were included. Additionally, illicit substance use over the last 3 months was examined by 3 cm-hair samples. The average concentration of each hair segment was calculated and used for the final analyses. Hair samples were analyzed for 512 drugs and their metabolites by an extremely sensitive and specific analytical technique–Liquid Chromatography Mass Spectrometry (LC-MS/MS). It is important to note that one participant in CG and two in PU did not self-report psychotropic medication which were detected in hair sample analysis, these participants were excluded from the study.

What is more 23.1% of PU (*n* = 6) did not report use of any other illicit psychoactive substance than cannabis, but we detected them in their hair sample analyses (see Supplementary Table 1).

### Procedure

Researchers collecting data were blind to the group status and had no knowledge of the cannabis/illicit drug use by the participants. Participants were asked to refrain from cannabis and other psychoactive substance use 12 h before attending the assessment session to ensure that examination would occur while they were not intoxicated. The abstinence was verified via the self-reported time and date of last use, and no observable signs of intoxication.

All participants were tested individually in one session. The experimenter showed the participant the lab and recording equipment and described the experimental protocol before written informed consent was obtained. Participants then completed a short demographics questionnaire and answered questions about their drug use in a separate room to protect their privacy. After that, subjects performed a shortened version of Wechsler Adult Intelligence Scale–Revised including five subtests (Wechsler, 1981; Brzeziński and Hornowska, 1998). Afterward, a 3 cm hair sample was taken from each participant (from the scalp).

Prior to the beginning of the experimental task, participants were verbally instructed as to what they would be experiencing and were shown what the procedure of EEG electrode mounting entails. Then participants were brought into a laboratory setting and seated in front of a 24 inch BenQ XL2411Z computer monitor (1,920 × 1,080 resolution, 100 Hz refresh rate) at a distance of 60 cm. Electrodes were then mounted and participants were briefly shown the EEG signal and explained how it is affected by eye blinks and muscular movements, which was a part of the procedure aimed at minimizing the amount of artifacts in the signal. The procedure was then started, and upon its completion subjects were provided with a place to wash their hair. The entire procedure lasted no more than 3 h.

#### Experimental Task

**FIGURE 1:**
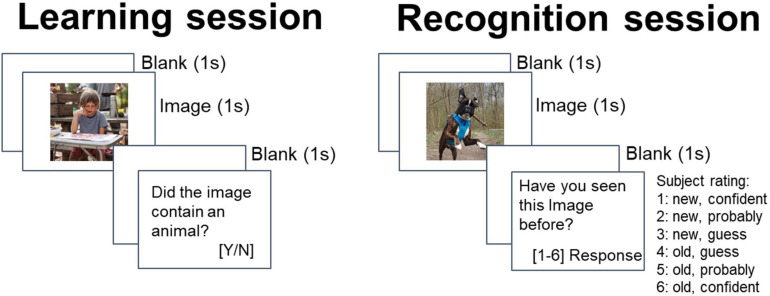
Trial structure at learning and recognition.

The episodic memory task consisted of two sessions: learning session and a recognition memory session at least 15 min later (Figure 1). During the learning session, participants viewed a set of 100 previously unseen, randomly selected images (each shown only once) from five categories, in random order (cars, people, animals, landscapes, food; from each category the same number of images was presented). Each image was presented for 1 s. After the image presentation, the subject assessed whether it showed an animal or not (the aim of that question was to maintain concentration on the task). The participants were informed that a test based on these images will be carried out later. Between the learning and recognition session there was at least a 15 min delay, during which a distraction task was administered (Sternberg) to prevent active rehearsal.

During the recognition session, participants viewed a set of 100 randomly selected images, 50 of which were new and 50 of which had been presented during the learning session (the same number of images were presented for each category). By using novel and complex pictures, we wanted to decrease chances of subjects to use verbalization as a memory strategy. If subjects had difficulty attaching a verbal label to the complex stimuli, then the probability of using this technique diminished. What is more, for the long delay period between learning and recognition phase (as in this study) information maintenance using a verbal strategy is not as efficient as in the case of immediate testing phase (Ellmore and Reichert, 2017). Subjects indicated whether they had seen the image before (response “old”) or not (response “new”) on a six-point confidence scale, along with the degree of confidence in their answer.

One hundred and fifty pictures were selected to be used in the current study from Wikimedia Commons under a Creative Commons license (stimuli from the Figure 1 have not been used in the study and come from the author’s private archive; examples of stimuli used in the study are in Supplementary Figure 1). The created database contains high quality photographs. All images were shown at the center of the screen of a computer placed in front of the participant. Participants responded by pressing marked buttons on a keyboard. There were no time restrictions for an answer. Stimulus presentation and recording of responses were attained using PsychoPy (v1.85.6; Peirce, 2007).

#### Signal Detection Theory

Performance during the memory task was analyzed with the SDT approach. An image correctly identified as old is a hit (remembered), an image incorrectly identified as old is a false alarm, an image incorrectly identified as novel is a miss (forgotten), and an image correctly identified as novel is a correct rejection.

The confidence levels in the ROC are cumulative and are calculated according to standard SDT analysis methods (Macmillan and Creelman, 2004). Each ROC curve was generated by plotting the hit rate against the false alarm rate for each confidence level of the 6-point confidence rating scale, from the most strict criterion (the proportion of hits and false alarms at the highest level of confidence) to the most liberal criterion, ending at (1,1). Recognition accuracy – the proportion of recognition (Pr) was calculated by subtracting false alarm rates from hit rates (Pr = H − FA) for each subject and was used as a measure of overall memory performance (Snodgrass and Corwin, 1988). Higher Pr values indicate better discriminability between old and new stimuli, which means better recognition accuracy.

The area under the ROC curve (AUC) is a measure of the discriminability, where 50% is chance and 100% is perfect discrimination. It is a reliable measure of performance accuracy as it is not influenced by response bias (C). *C* value (*C* = −0.5[(*z*(H) + *z*(FA)]) is a decision bias parameter. Positive C values indicate conservative response bias, while negative indicate liberal response bias. The other measure of discriminability or sensitivity is *d*-prime [*d*’ = *z*(H) - *z*(FA)] which usually refers to the degree to which latent memory or perceptual signals from two classes of repeatedly presented stimuli–target stimuli or lure stimuli–overlap in the brain. Statistic *d*’ is the standardized distance between the means of two underlying strength distributions that are assumed to be Gaussian in form and to have equal variance and take values between 0 and 4 SD. If those two distributions overlap completely, then discriminability is equal to zero. Detection capability/sensitivity (*d*’) increases as the number of hits increases and as the number of false alarms decreases.

#### EEG Recordings and Analysis

A 64-channel SynAmps RT Neuroscan EEG amplifier and BrainProducts actiCap Ag/AG–Cl active electrode set were used to record brain activity during task performance. All channels were recorded at 1000 Hz sampling rate. Impedances were held below 15 kΩ. All data was preprocessed offline using MATLAB environment, EEGlab (Delorme and Makeig, 2004), and ERPlab (Lopez-Calderon and Luck, 2014) software packages. The signal was initially re-referenced to a common average and then down-sampled to 250 Hz, followed by a high-pass filter (cut-off = 0.1 Hz). Signals from bad electrodes were interpolated. Movement artifacts were manually removed from the data, after which an independent component analysis (ICA) was applied for an eyeblink artifact rejection. Data epochs between −200 and 996 ms (with zero being the image presentation) were extracted. The epochs were visually inspected for remaining eye-blinks/movements and excessive muscle activity. Then the segments were averaged from trials with correct responses, according to the condition [(i) New image, (ii) Old image]. Correct old trials (Old image) were calculated for old items rated 5 or 6, while correct new trials (New image) were calculated for new items rated 1 or 2. Incorrect responses were not included in analyses as there were not enough of them. Baseline correction of ERP amplitudes was performed for the interval from -200 to 0 ms.

**FIGURE 2:**
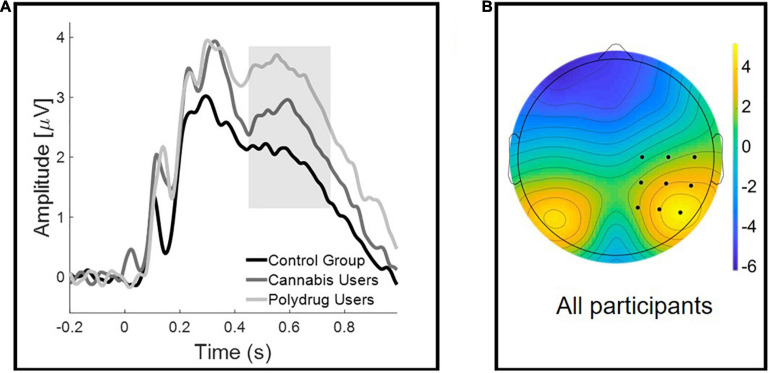
Group differences in the Late Positive Component (LPC). **(A)** Grand-averaged waveforms at a representative right centro-occipital cluster for correctly recognized images. The shaded area represents the late (450–750 ms) time window used for the analyses. **(B)** Scalp topographies of the mean activity in 450–750 ms time window.

The FN component was measured from the 350–550 ms time window, from the F3, Fz, and F4 electrodes. LPC time window was specified to 450–750 ms, and was obtained from electrodes: C2, C4, C6, CP2, CP4, CP6, P2, P4, and P6 (Figure 2B). ERPs’ time windows and electrodes were defined according to the literature and corresponding topographical maps (Rugg and Curran, 2007; Danker et al., 2008; Hoppstädter et al., 2015; Tsivilis et al., 2015; Kamp et al., 2016).

All analyses were conducted using IBM Corp. Released 2017. IBM SPSS Statistics for Windows, Version 25.0. Armonk, NY: IBM Corp. and MATLAB custom scripts. An alpha level of 0.05 was used for all statistical tests.

## Results

### Group Characteristics

Group comparisons for demographic, general functioning, intelligence quotient and substance use were conducted with a series of *t*-tests or ANOVAs for continuous variables, Mann–Whitney *U* or Kruskal–Wallis *H* tests for most substance use measures (on ordinal scales) and a χ^2^-test for categorical variables. There were no significant differences between groups in age, sex, education years, self-reported diagnosed psychiatric disorders, verbal and fluid intelligence, alcohol and tobacco use patterns nor for groups included in behavioral analyses nor in ERPs analyses as shown in Tables 1, 2, respectively. Fluid intelligence was assessed using Matrix Reasoning and Block Design from WAIS-R. Verbal intelligence was assessed using Vocabulary, Similarities and Digit Span from WAIS-R.

There were no differences in age of cannabis use onset, frequency of cannabis use, dose in grams per week, frequency of cannabis use within the past 30 days and dose in puffs per one use between CU and PU (see Tables 1, 2).

### Behavioral Performance

Memory performance estimates were calculated using the ROC toolbox for MATLAB (Koen et al., 2017). Table 3 lists memory performance indices for three groups. One-way ANOVA was computed for each of the behavioral indices to test for significant differences. There were no differences in *H* [*F*(2, 76) = 0.472, *p* = 0.626, η^2^ = 0.12], FA [*F*(2, 76) = 1.510, *p* = 0.228, η^2^ = 0.038], Pr [*F*(2, 76) = 0.159, *p* = 0.854, η^2^ = 0.004], *d*’ [*F*(2, 73) = 0.238, *p* = 0.789, η^2^ = 0.006], AUC [*F*(2, 76) = 0.648, *p* = 0.526, η^2^ = 0.017], and c [*F*(2, 73) = 1.574, *p* = 0.214, η^2^ = 0.041] between groups. The statistically insignificant differences were preserved when we excluded four participants who reported last use less than 12 h before testing from analyses.

**TABLE 3:** Recognition memory performance – mean (SD).

	**Control group**	**Cannabis users**	**Polydrug users**
Hits (*H*)	0.78 (0.11)	0.77 (0.12)	0.75 (0.11)
False alarms (FAs)	0.12 (0.08)	0.1 (0.06)	0.09 (0.06)
**Discrimination indices**			
Pr	0.66 (0.13)	0.68 (0.13)	0.66 (0.12)
*d*’	2.08 (0.58)	2.19 (0.51)	2.12 (0.5)
AUC	0.89 (0.59)	0.89 (0.57)	0.88 (0.62)
**Response bias indices**			
C	0.2 (0.31)	0.25 (0.27)	0.35 (0.29)
**Reaction time (RT)**			
Old correct RT [s]	0.672 (0.3)	0.741 (0.21)	0.639 (0.27)
New correct RT [s]	0.681 (0.34)	0.728 (0.21)	0.646 (0.44)

*Note Reaction times reported in seconds [s].*

**FIGURE 3:**
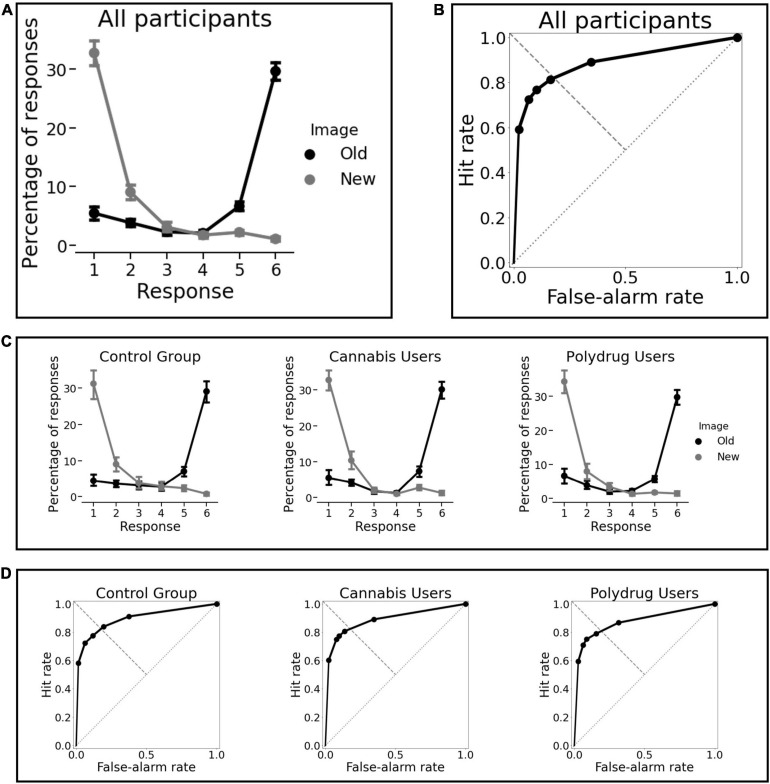
Behavioral results. **(A)** Percentage of responses “old” and “new” as a function of confidence for all participants As *X*-axis corresponds to participants’ rating (1 = new, confident; 6 = old, confident) the highest probabilities for responses Old and New were 6 and 1. **(B)** The average receiver operating characteristic of all subjects. **(C)** Percentage of responses “old” and “new” as a function of confidence for each group separately. **(D)** The average receiver operating characteristic for each group separately. There were no significant differences between groups for any memory performance indicator and we can observe obvious similarities in memory performance across groups.

Participants successfully indicated the presence or absence of an animal in 94% of the trials, which means they were focused on the task. In general, subjects performed well. As shown in Table 3 the average sensitivity (*d*’) and recognition accuracy (Pr) were high. Participant’s reports of confidence fit well to their performance (Figures 3A,C) and the ROC were assymetrical (Figures 3B,D) with high average AUC values. Only one participant AUC was 0.58, while 0.5 indicated performance at a random level, this subject was excluded from the study due to deviant results and lack of engagement in the task.

We also compared reaction times between correct old and correct new trials. Correct old trials were calculated for old items rated 5 or 6, while correct new trials were calculated for new items rated 1 or 2, the same as in EEG analyses. Reaction times were individually standardized and the observations above the three standard deviations (for each participant separately) were excluded from data analysis. RT for each group separately is reported in Table 3. Repeated-measures ANOVAs, with Old/New (two levels: Old Image vs. New Image) as a within-participant factor, and Group (three levels: CG vs. CU vs. PU) as a between-participant factor, were performed on the reaction times. ANOVA analysis revealed that there was neither a main effect of Old/New [*F*(1, 76) = 0.002, *p* = 0.962, η^2^ = 0.00] nor the Old/New × Group interaction [*F*(2, 76) = 0.078, *p* = 0.925, η^2^ = 0.002]. All effects for RT remain statistically insignificant when we excluded four participants who reported last use less than 12 h before testing from analyses.

### Electrophysiological Data

#### Frontal Negativity

**FIGURE 4:**
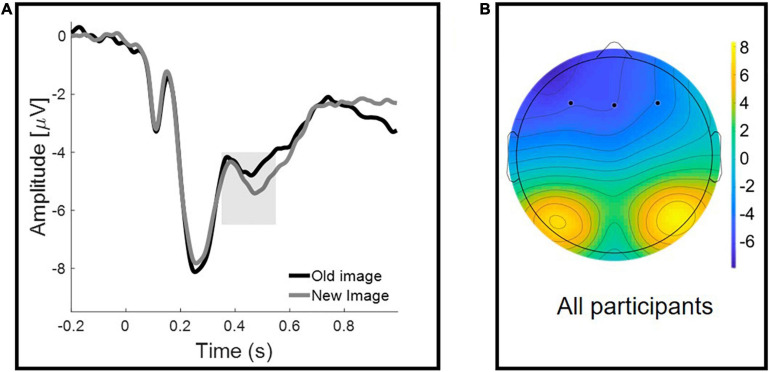
Frontal Negativity for Old/New effect reflecting familiarity. **(A)** Grand-averaged waveforms at a representative frontal cluster for correctly recognized old and new images. The shaded area represents the early (350–550 ms) time window used for the analyses. **(B)** Scalp topographies of the mean activity in 350–550 ms time window.

Figure 4A illustrates the grand average ERPs for correctly recognized old and new images. Repeated-measures ANOVA, with Old/New (two levels: Old Image vs. New Image) as a within-participant factor, and Group (three levels: CG vs. CU vs. PU) as a between-participant factor, was performed on the mean FN amplitudes. ANOVA analysis revealed that there was a significant Old/New effect [*F*(1, 60) = 5.211, *p* = 0.026, η^2^ = 0.08] but no Group effect [*F*(2, 60) = 0.35, *p* = 0.706, η^2^ = 0.012], nor Old/New × Group interaction [*F*(2, 60) = 0.019, *p* = 0.981, η^2^ = 0.001].

Similarly, analysis, which excluded participants with shorter than 12 h abstinence since last cannabis use, showed significant Old/New effect [*F*(1, 57) = 5.882, *p* = 0.018, η^2^ = 0.094] and no Group effect [*F*(2, 57) = 0.305, *p* = 0.738, η^2^ = 0.011], nor Old/New × Group interaction [*F*(2, 57) = 0.032, *p* = 0.969, η^2^ = 0.001]. Specific values of the FN are presented in Table 4.

**TABLE 4:** Means and standard deviations of the mean amplitudes (μV) of the Frontal Negativities for all groups and conditions.

	**All participants**			**Excluding participants with shorter than**	
				**12 h abstinence since last cannabis use**	
	**Control group**	**Cannabis users**	**Polydrug users**	**Cannabis users**	**Polydrug users**
Old image	-3.92 (3.60)	-4.80 (4.38)	-4.54 (3.26)	-4.61 (4.62)	-4.65 (3.31)
New image	-4.45 (3.26)	-5.28 (3.69)	-4.98 (2.95)	-5.23 (3.90)	-5.12 (2.95)
Mean	-4.16 (3.35)	-5.04 (3.97)	-4.74 (3.00)	-4.90 (4.20)	-4.88 (3.01)

#### Late Positive Component

Figure 2A illustrates the mean amplitudes of correctly recognized images for each group. As mentioned before, we did not include data for incorrectly recognized images in the analysis.

Repeated-measures ANOVA, with Old/New (two levels: Old Image vs. New Image) as a within-participant factor, and Group (three levels: CG vs. CU vs. PU) as a between-participant factor, was performed on the mean LPC amplitudes. ANOVA analysis revealed that there was a significant Group effect [*F*(2, 60) = 20.478, *p* = 0.017, η^2^ = 0.127] but no main effect of Old/New [*F*(1, 60) = 0.706, *p* = 0.404, η^2^ = 0.012], nor Old/New × Group interaction [*F*(2, 60) = 1.336, *p* = 0.271, η^2^ = 0.043].

Bonferroni-corrected multiple comparisons revealed significant difference between CG and PU (*p* = 0.013) but not between CG and CU (*p* = 0.593) or CU and PU (*p* = 0.425).

Analysis, which excluded participants with shorter than 12 h abstinence since last cannabis use, showed significant Group effect [*F*(2, 57) = 3.961, *p* = 0.025, η^2^ = 0.122] and almost significant Old/New × Group interaction [*F*(2, 57) = 2.693, *p* = 0.076, η^2^ = 0.086], but no main Old/New effect [*F*(1, 57) = 0.918, *p* = 0.342, η^2^ = 0.016]. Bonferroni-corrected multiple comparisons revealed significant difference between CG and PU (*p* = 0.02) but not between CG and CU (*p* = 0.571) or CU and PU (*p* = 0.636).

As previous studies have reported, LPC is usually lateralized on the left centro-occipital sphere (Curran and Cleary, 2003; Rugg and Curran, 2007), we decided to duplicate presented analyses on the left-lateralized electrode cluster (electrodes: C1, C3, C5, CP1, CP3, CP5, P1, P3, and P5). Then it was revealed that neither Group difference [*F*(2, 60) = 1.174, *p* = 0.317, η^2^ = 0.038], Old/New effect [*F*(1, 60) = 1.815, *p* = 0.183, η^2^ = 0.029], nor Old/New × Group interaction [*F*(2, 60) = 0.121 *p* = 0.886, η^2^ = 0.004] were significant on the left-lateralized cluster.

Specific values of the LPC are presented in Table 5.

**TABLE 5:** Means and standard deviations of the mean amplitudes (μV) of the Late Positive Components for all groups and conditions.

	**All participants**			**Excluding participants with shorter than**	
				**12 h abstinence since last cannabis use**	
	**Control group**	**Cannabis users**	**Polydrug users**	**Cannabis users**	**Polydrug users**
Old image	2.01 (1.75)	2.71 (1.82)	3.22 (1.45)	2.81 (1.91)	3.16 (1.45)
New image	1.88 (1.63)	2.42 (1.45)	3.37 (1.36)	2.41 (1.45)	3.38 (1.40)
Mean	1.95 (1.67)	2.57 (1.55)	3.30 (1.33)	2.61 (1.61)	3.27 (1.36)

#### Regression Models

We did not observe any significant differences in any of the memory performance indices between groups, which was also observed in some previous studies (Smith et al., 2017; Sagar and Gruber, 2019). While the Old/New effect was not observed for the LPC component, there were significant differences in mean amplitude between groups in this component for correct answers. As LPC reflects attention, motivation, higher cognitive function, decision accuracy, and memory judgments (MacNamara et al., 2011; Wiens and Syrjänen, 2013; Ratcliff et al., 2016; Yang et al., 2019), we decided to look for its influence on the measure of overall memory performance – recognition accuracy (Pr). To test whether the mean amplitude of the LPC component predicts participants’ Pr, a linear regression was calculated.

**FIGURE 5:**
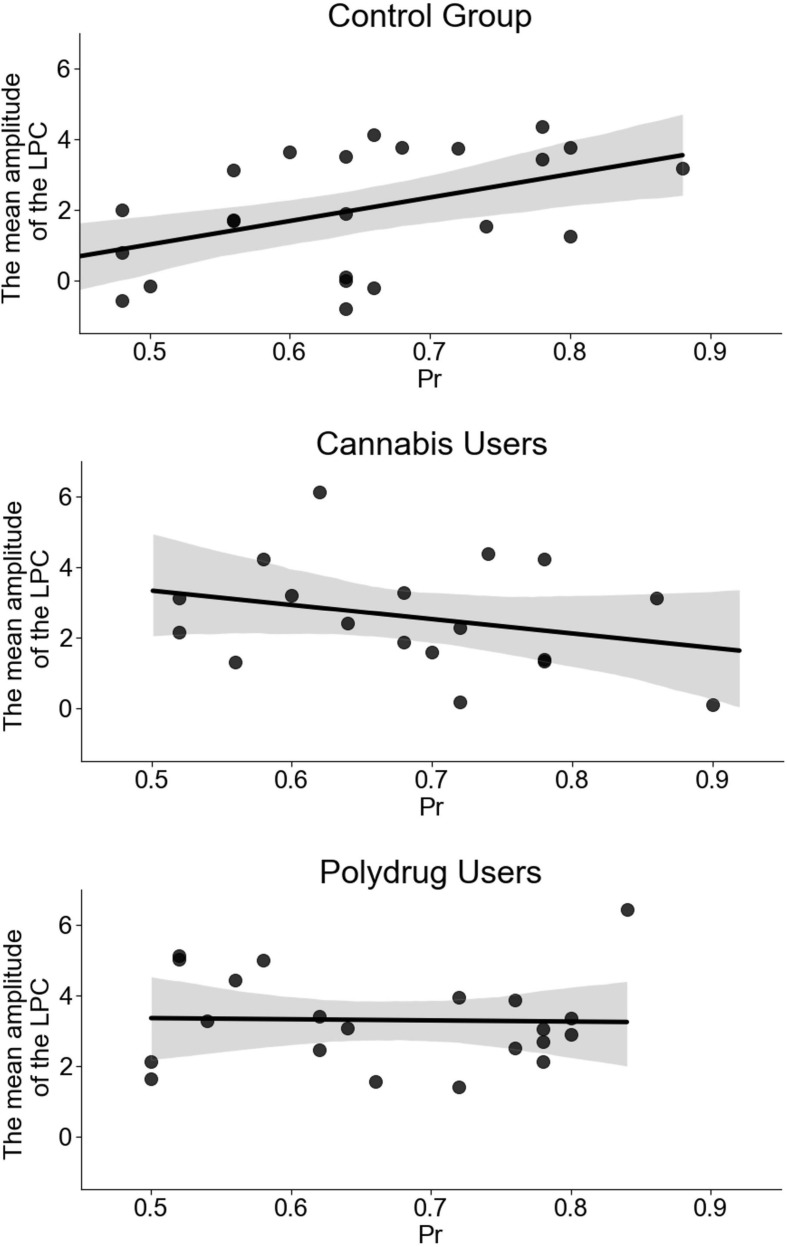
Regression models for recognition accuracy indicator (proportion of recognition, Pr = Hits - False Alarms) as a function of the mean amplitude of the LPC for each group separately. Scatter plot presents a significant relation between the mean amplitude of the LPC and Pr (*r* = 0.472, *p* = 0.02) in control group (CG) and statistically insignificant relations in cannabis users (CU) (*r* = −0.287, *p* = 0.248) and polydrug users (PU) (*r* = −0.028, *p* = 0.903).

While the initial model, containing three groups, turned out to be statistically insignificant [*F*(3, 59) = 1.902, *p* = 0.139] with an R^2^ = 0.088, it contained significant influence of the mean LPC amplitude (*b* = 0.027, *p* = 0.043), interaction between the amplitude and the Group (*b* = −0.023, *p* = 0.045), and almost significant influence of the Group (*b* = 0.07, *p* = 0.05). As the insignificance of the initial model may be a result of an actual relation existing in only one group, we decided to look at each group independently (see Figure 5). Then it was revealed that while the influence of the LPC was significant in CG (*b* = 0.034, *p* = 0.02), both CU (*b* = −0.02, *p* = 0.248) and PU (*b* = −0.002, *p* = 0.903) models were statistically insignificant.

Similarly, a model computed with extension of participants with abstinence shorter than 12 h since last cannabis use was statistically insignificant [*F*(3, 56) = 2.104, *p* = 0.11, R2 = 0.101], but contained significant influence of the mean LPC amplitude (*b* = 0.028, *p* = 0.036), Group (*b* = 0.077, *p* = 0.037) and interaction between those (*b* = -0.025, *p* = 0.028). Auxiliary models, created for each of groups independently, presented similar results (CG: *b* = 0.034, *p* = 0.02; CU: *b* = −0.023, *p* = 0.156; PU: *b* = −0.004, *p* = 0.844).

## Discussion

We believe this is the first EEG investigation of prolonged THC exposure on memory processes in regular cannabis users and regular cannabis polydrug users. The results showed alterations in recognition memory processing, which were not manifested on a behavioral level, but were prominent on a physiological level and expressed the most in cannabis polydrug users. Based on the experimental procedure used in our study and calculated behavioral indicators, we were able to precisely analyse participants’ performance. Subjects’ confidence rating charts and receiver operating characteristics, averaged for each group separately (Figure 3) indicate they had an overall good quality of their memories. It is also a signature that MTL-dependent declarative memories were formed successfully (Wais et al., 2006).

The results have shown significant differences in mean LPC amplitudes for correct answers between CG and PU. As mentioned before the LPC is not unique for episodic memory recollection, but is engaged in decision making during memory judgments and related to various cognitive functions (Brezis et al., 2016; Ratcliff et al., 2016). Increased brain activity during cognitive task could be interpreted as a compensational, stronger “neurophysiological” effort to overcome drug-induced brain dysfunction and maintain normal behavioral performance (Zeineh et al., 2003; Bhattacharyya et al., 2009; Roberts et al., 2009), possibly higher engagement of attentional and motivational resources in this case.

It is quite surprising, however, that we have not found significant differences in LPC between CU and CG or PU. The possible explanation is that use of other illicit drugs among cannabis users altered brain function much more than cannabis use only, and the differences observed between the control group and cannabis users in previous studies were enhanced by co-occurring use of other illicit drugs. The most popular other illicit psychoactive substance among PU in our study was MDMA. As previous studies have shown MDMA is the most commonly used drug in polydrug context with cannabis (Scholey et al., 2004; Daumann et al., 2004; European Monitoring Centre for Drugs and Drug Addiction (EMCDDA), 2018). MDMA acts via serotonergic receptors and may cause disturbances of serotonergic pathways (Benningfield and Cowan, 2013) and electrophysiological changes that reflect recruitment of additional resources to perform cognitive tasks (Roberts et al., 2018). The other psychoactive substances used among PU, such as cocaine, amphetamine, and others may interact in a complex way and cumulative effect may impact the further increased brain activity (Zilverstand et al., 2018; Khajehpour et al., 2019). Still, little is known about the interactive impact of various illicit drugs on the human brain (Gouzoulis-Mayfrank and Daumann, 2006; Van Dam et al., 2008; Shevlin et al., 2017), regardless of the observed high frequency of polydrug use pattern.

What is more, the age of cannabis use onset in our participants (∼20) could play an important role in shaping our results (no difference between CU and CG), as usually in cannabis research the age of onset is lower (during adolescence). That is why, our results may be not possible to generalize on cannabis users that started regular cannabis use much earlier. At the same time, our participants were middle-aged and have shown long lifetime cannabis use (∼10 years), giving us the opportunity to investigate higher cumulative effects of cannabis use. It is worthy to note that Smith et al. (2017) observed larger LPC (indexing recollection) in heavy drinkers compared to a control group, but not in cannabis users, without any concomitant behavioral impairments.

We have also examined the direct relationship between the ERP components’ amplitude and memory performance. We checked whether the value-modulated LPC predicted behavioral measures of a participant’s recognition accuracy. The initial regression model containing LPC × Group interaction was statistically insignificant, but further exploration revealed that LPC amplitude predicts recognition accuracy in the control group only. This result suggests that the higher the involvement of the processes reflected in the LPC, the better non-drug using participants are in discriminating between old and new stimuli. Previous study has shown a positive correlation between performance level on recognition memory task and LPC amplitude only in healthy older people, but it was not observed in participants suffering from mild cognitive impairment (Waninger et al., 2018).

Due to a small CU sample size in our study, this explanation should be treated with caution and should be further explored in a larger sample. It is important to note that LPC is located predominantly over posterior sites, often but not always exhibiting a left-sided maximum, while in our study it showed a right-sided maximum. It is not common but was observed before (Tanguay et al., 2018; Hebscher et al., 2020).

Consistent with previous studies, we have observed a frontal old/new effect thought to index familiarity, ERPs elicited by previously seen stimuli (Old Images) were significantly more positive in all groups (Curran, 2000; Paller et al., 2007). However, we did not observe a parietal old/new effect (LPC). The absence of LPC mean amplitude changes, depending on whether stimuli was correctly identified as old or new, has been reported in the literature before (Danker et al., 2008; Wolk et al., 2009; Addante et al., 2012) and could be interpreted as reduction in recollective processes following successful familiarity (Tibon and Henson, 2015; Kamp et al., 2016). The FN400 and LPC are usually interpreted in the context of the dual-process model of recognition memory, where the LPC is the index of recollection of episodic details about the prior stimulus encounter, and the FN400 marks item-based, context free, familiarity assessment (Yonelinas, 2002). It should be mentioned that there is a still ongoing debate on the interpretation of these two ERP components of recognition memory and the processes that underlie them (Voss and Federmeier, 2011; Bridger et al., 2012).

However, what was a little bit surprising for us, we did not see any visible differences in behavioral indices of visual episodic memory between groups. In light of our results, it should be noted that studies employing neuroimaging and electrophysiological techniques proved to be more sensitive to detect drug effects (especially in non-intoxicated users) than traditional, behavioral measures (Jager et al., 2007; Nestor et al., 2008; Cousijn et al., 2014). Altered brain activation patterns in cannabis users relative to non-users were observed across numerous brain regions even when no differences in task performance were detected (Smith et al., 2017; Sagar and Gruber, 2019).

In line with these results, it is interesting to note that LPC amplitude in general was higher in CU and PU (Figure 2A), but it did not predict memory accuracy. It may reflect subtle alterations in neural circuits engaged in memory processing in both CU and PU. Possibly, LPC rather reflects compensatory mechanisms mentioned before, and higher attentional and/or motivational engagement in PU and CU, as it does not correspond to a recognition accuracy indicator in this case.

### Strengths and Limitations

The use of hair sample analysis to define groups exposed or not to cannabis and other drugs is limited in that it only provides information on exposure over the prior 3 months (when 3 cm of hair is analysed). Unfortunately, hair sample analysis was not available for eight participants from the control group. However, it is important to note that biological measures of drug use (illicit substances or psychotropic medication) is rarely used in non-using controls in the research field on cannabis (or other illicit drugs) and self-reported measures dominate for CU (Smith et al., 2017; Khajehpour et al., 2019; Rangel-Pacheco et al., 2020) or the urine/hair sample analyses detect cannabis use only (no other illicit drugs nor psychotropic medication) (Yücel et al., 2016; Prashad et al., 2018). That is why we would consider it as an additional value of our study and exclusion of this participants form analyses would make CG too small to obtain reliable results. What is more, the obtained memory performance results and ERPs are in line with previous studies and do not show any artifacts (which possible drug use in this group could generate). The reason why hair samples were not collected in this group was that participants did not want to lose a big amount of hair (diameter of a pencil) required for hair sample analyses, because of esthetic reasons. As the observed tendency among participants was rather to under-report drug use in cannabis users (mainly in case of other illicit drug use) we assumed their self-reports were reliable. In general, the best practice is to use hair analysis with complementary tests for urine and blood analysis as THC (similar to other drugs) takes about 2 weeks to reach the hair shaft (Shah et al., 2019). Unfortunately, we did not use these measurements in our study, because of funding limitations and ethical concerns. At the same time, combined self-reported and objective hair sample analyses were also the strength of our study. While there is growing popularity of using a combination of self-report and objective drug use assessment in research on cannabis and neurocognitive functioning, the most popular are drug urine tests, which despite all advantages have a relatively short time-frame for various illicit drug detection. The hair sample analysis provides the opportunity to detect many drugs metabolites among much longer time-frames (restricted by subjects’ hair length and research funding limits), which makes it a suitable tool for long-term drug use assessment. This kind of analysis has been used in research on cannabis and neurocognition, but it has been restricted to cannabinoid metabolites detection only. While hair sample analyses did not prove THC presence in all cannabis users, it allowed to exclude other illicit drug use in CU. Previous research showed that the sensitivity of THC detection in hair is almost 80% in heavy cannabis smokers compared to light and non- cannabis users, but fell to 55% in any cannabis users compared to non-cannabis users (Taylor et al., 2017).

The highly heterogeneous PU group is an important limitation in our study (Supplementary Table 1), because we did not include it preliminary in our research plan (as described in “Participants” section). However, we decided to include them in a study as a separate group (PU) as constituting a representative sample of cannabis users (Mitchell and Plunkett, 2000; Carlson et al., 2005; Lynskey et al., 2006; World Health Organisation, 2016). That is why we did not collect detailed information about the polydrug use pattern (e.g., frequency of use, lifetime use, whether different substances were used concurrently or sequentially, substance dependence) as we intended to recruit cannabis users only. This information would be beneficial to understand better the issues of polydrug use and select a more homogeneous group in future studies.

Moreover, our measure of psychiatric symptoms is based exclusively on self-report (self-declaration of the presence of a diagnosis by a mental health specialist), rather than clinical evaluation or a structured interview. It would be important to engage medical professionals in future studies for psychiatric diagnosis as it is an important factor in polydrug use context. While we used CUDIT-R to screen the severity of use-related problems and recruit only participants with negative results in screening for cannabis use disorder, we did not include in our study screening for the severity of other illicit drug use-related problems.

The control group in our study did not include only individuals who had never used cannabis, but some of them reported minimal use in their lifetime (<50 occasions). It is considered acceptable and attenuates a potential cumulative effect of cannabis use (Sagar and Gruber, 2019). We are aware of the modest group sizes in our study, however, most neuroimaging investigations in cannabis research have similar sample sizes (Sagar and Gruber, 2019). While these samples appear to be large enough to detect between-group differences in brain activation patterns, it should be noted that our study has preliminary character and further research is needed.

In summary, the findings of the present study indicate that, when patterns of cannabis and polydrug use are examined in greater detail, the unique effect of cannabis consumption seems to be greatly attenuated. There was no significant effect for cannabis alone, but only for cannabis in conjunction with other illicit drugs, which most likely produce the biggest disturbance in brain function.

## Data Availability Statement

The raw data supporting the conclusions of this article will be made available by the authors, without undue reservation.

## Ethics Statement

The studies involving human participants were reviewed and approved by the SWPS University Research Ethics Committee. The patients/participants provided their written informed consent to participate in this study. Written informed consent was obtained from the minor(s)’ legal guardian/next of kin for the publication of any potentially identifiable images or data included in this article.

## Author Contributions

AAB, and AB: study the design. AAB, NJ, and MG: data processing and data analysis. NG and AP-C: data analysis. AAB, AB, NJ, MG, NG, and AP-C: writing the manuscript. All authors contributed to the article and approved the submitted version.

## Conflict of Interest

The authors declare that the research was conducted in the absence of any commercial or financial relationships that could be construed as a potential conflict of interest.
