# Supplementary material for: Not Just a Pot: Visual Episodic Memory in Cannabis Users and Polydrug Cannabis Users: ROC and ERP Preliminary Investigation
Source: Front Hum Neurosci. 2021 Jun 11;15:677793. doi: 10.3389/fnhum.2021.677793 (PMC8226271; doi:10.3389/fnhum.2021.677793)
Supplement: Supplementary file 1 [file Data_Sheet_1.PDF]

Supplementary Material

Supplementary table 1. Detailed characteristics of polydrug users (PU): self-reported illicit drug use (other than cannabis) and hair sample analyses results

Participant	Hair sample analysis results	Self-reported other illicit drug use in last 3 months	Self-reported other illicit drug use in last 30 days
1	MDMA	cannabis, LSD, MDMA,DMT, amphetamine	0
2	MDMA	cannabis, amphetamine	1 time per month
3	MDMA	MDMA, cannabis	1 time per month
4	MDMA	MDMA, cannabis	0
5	MDMA, cocaine,THC	MDMA, cannabis, cocaine, amphetamine	1 time per month
6	MDMA, amphetamine, THC	MDMA, cannabis	1 time per month
7	MDMA, cocaine, THC	cannabis, cocaine	1 time per month
8	MDMA, THC	cannabis, MDMA, psilocybin	0
9	MDMA, THC	LSD, cannabis, MDMA, amphetamine	0
10	MDMA, THC	MDMA, cannabis	0
11	MDMA, THC	cannabis	1 time per month
12	MDMA, THC	cannabis, novel psychoactive substance	0
13	MDMA, cocaine	cannabis	1 time per month
14	amphetamine	cannabis	0
15	amphetamine, MDMA, THC	amphetamine, MDMA, cannabis	1 time per month
16	amphetamine, cocaine, THC,	cocaine, cannabis	0
17	cocaine, THC	cannabis	0
18	cocaine,THC	cannabis	0
19	cocaine,THC	cocaine, cannabis, novel psychoactive substance	1 time per month
20	cocaine, LSD, THC	LSD, cannabis	0
21	methcathinone, THC	0	0
22*	cocaine, THC	cocaine, cannabis	0

23*	cathine, methcathinone, THC	cannabis	0
24*	MDMA	MDMA, psilocybin	0
25*	THC, MDMA	MDMA, psilocybin, cannabis	2< per month
26*	MDMA, THC	cannabis	0

Note: Participants in PU (n = 26) included in behavioral data analyses, while 21 were included in ERPs analyses (5 participant were excluded because of bad EEG signal; marked with *).

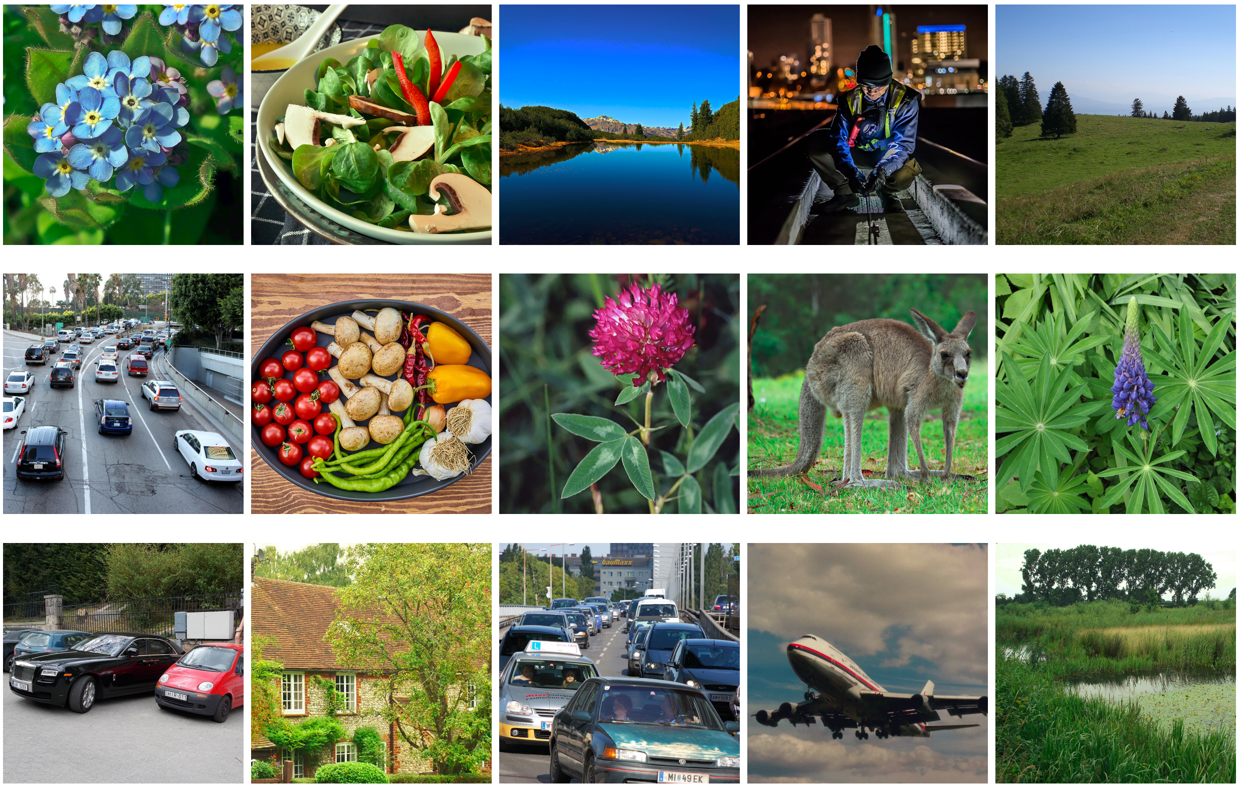

SFig. 1 Examples of the stimuli used in memory task. Stimuli come from 5 different visual categories: people, food/flowers, cars/vehicles, animals and outdoor scenes/houses. Images are not shown to scale.
